# High incidence of (osteo)chondral lesions in ankle fractures

**DOI:** 10.1007/s00167-020-06187-y

**Published:** 2020-08-06

**Authors:** Hugo A. Martijn, Kaj T. A. Lambers, Jari Dahmen, Sjoerd. A. S. Stufkens, Gino M. M. J. Kerkhoffs

**Affiliations:** 1 Department of Orthopedic Surgery, Location AMC, Amsterdam Movement Sciences, Amsterdam UMC, University of Amsterdam, Amsterdam, The Netherlands; 2 Academic Center for Evidence Based Sports Medicine (ACES), Amsterdam, The Netherlands; 3 Amsterdam Collaboration for Health and Safety in Sports (ACHSS), AMC/VUmc IOC Research Center, Amsterdam, The Netherlands; 4 Department of Orthopedic Surgery, Amphia Hospital, Breda, The Netherlands

**Keywords:** “Osteochondral lesion”, “Ankle”, “Ankle fracture”

## Abstract

**Purpose:**

To determine the incidence and location of osteochondral lesions (OCLs) following ankle fractures as well as to determine the association between fracture type and the presence of OCLs. Up to 50% of patients with ankle fractures that receive surgical treatment show suboptimal functional results with residual complaints at a long-term follow-up. This might be due to the presence of intra-articular osteochondral lesions (OCL).

**Methods:**

A literature search was carried out in PubMed (MEDLINE), EMBASE, CDSR, DARE and CENTRAL to identify relevant studies. Two authors separately and independently screened the search results and conducted the quality assessment using the MINORS criteria. Available full-text clinical articles on ankle fractures published in English, Dutch and German were eligible for inclusion. Per fracture classification, the OCL incidence and location were extracted from the included articles. Where possible, OCL incidence per fracture classification (Danis–Weber and/or Lauge–Hansen classification) was calculated and pooled. Two-sided *p* values of less than 0.05 were considered statistically significant.

**Results:**

Twenty articles were included with a total of 1707 ankle fractures in 1707 patients. When focusing on ankle fractures that were assessed directly after the trauma, the OCL incidence was 45% (*n* = 1404). Furthermore, the most common location of an OCL following an ankle fractures was the talus (43% of all OCLs). A significant difference in OCL incidence was observed among Lauge–Hansen categories (*p* = 0.049). Post hoc pairwise comparisons between Lauge–Hansen categories (with adjusted significance level of 0.01) revealed no significant difference (n.s.).

**Conclusion:**

OCLs are frequently seen in patients with ankle fractures when assessed both directly after and at least 12 months after initial trauma (45–47%, respectively). Moreover, the vast majority of post-traumatic OCLs were located in the talus (42.7% of all OCLs). A higher incidence of OCLs was observed with rotational type fractures. The clinical relevance of the present systematic review is that it provides an overview of the incidence and location of OCLs in ankle fractures, hereby raising awareness to surgeons of these treatable concomitant injuries. As a result, this may improve the clinical outcomes when directly addressed during index surgery.

**Level of evidence:**

IV.

## Introduction

Ankle fractures are common injuries, with a global annual incidence of 0.1–0.2% [49, 53, 54]. Operative treatment focuses on achieving stability, anatomic reduction and congruity of the ankle joint by means of open reduction and internal fixation. Up to 50% of the surgically treated patients show suboptimal functional results with residual complaints at long-term follow-up [21, 41, 47, 52]. A frequent residual complaint is a persistent pain, which can have a large impact on the daily-functioning of patients [40]. One of the potential explanations for this residual pain could be the presence of (osteo)chondral lesions (OCLs) thereby impeding the clinical recovery of the individual patients [32, 49, 53, 54].

When studying the relationship between ankle fractures and the presence of OCLs in the ankle, it becomes clear that there is a substantial lack of knowledge on (1) the exact incidence of these lesions in ankle fractures, (2) the location of these OCLs and (3) the association between OCLs and ankle fracture type.

Although no exact incidence of OCLs after ankle fracture is known, incidences in the literature range from 10% to almost 90% [28, 42]. A post-traumatic talar OCL is thought to occur when the talus is rotated or translated in the loaded ankle mortise until the fracture occurs [28]. As demonstrated by Bruns et al. [9] in cadaveric ankle joints, the maximum pressure on the lateral talar border was observed in valgus and pronation position, whereas trauma in supination stresses the medial half of the ankle joint [28]. Raikin et al. [45] confirmed this in a large study in which medial talar OCL incidence was 63%. The high incidence could be explained because many OCLs are related to inversion injuries and could, therefore, result in an impaction of the medial talar dome. Verhagen et al. [57] reported an incidence of 61% of medial dome OCL after ankle trauma.

Furthermore, conflicting findings have been reported concerning the association between ankle fracture type and the incidence of OCLs in the ankle. For instance, Hintermann et al. [28] described that the frequency and severity of the lesions significantly increased from type-B to type-C fractures (classification according to AO-Danis-Weber [19, 58]), whereas Nosewicz et al. [42] found no significant association between these fracture types. Regier et al. [46] illustrated that patients with trimalleolar fractures or dislocated ankle fractures had a significantly higher risk of developing an OCL compared to patients with unimalleolar type B fractures.

The discrepancies between the scarce amount of evidence make it clear that the exact incidence of OCLs in ankle fractures is not yet known. This also holds for the exact location of post-fracture OCLs, as well as the association between OCLs and the severity of ankle fracture types. Therefore, we hypothesized that the incidence of OCLs is higher in rotational type ankle fractures. To the best of our knowledge, no previous systematic review has been published studying the before-mentioned. Therefore, the aim of the present study is to systematically review the current literature to determine the OCL incidence after ankle fractures, to determine the most common location, and, finally, to determine the association between OCLs and fracture type. If concomitant OCLs in acute ankle fractures are correctly diagnosed and treated accordingly, this may improve the clinical and functional outcome after surgery.

## Materials and methods

The PRISMA statement (Preferred Reporting Items for Systematic reviews and Meta-Analyses) was used as a guideline for the present study. The protocol for our systematic review and meta-analysis was prospectively registered in the PROSPERO register with registration number CRD42018086653 [16].

### Search strategy

PubMed (MEDLINE), EMBASE, CDSR, DARE and CENTRAL were used for a systematic search performed in May 2019 to identify potentially suitable studies. Backward citation chaining strategy was used to identify additional eligible studies. The full search strategy can be found in (Appendix I).

### Eligibility criteria and study selection

**Fig. 1:**
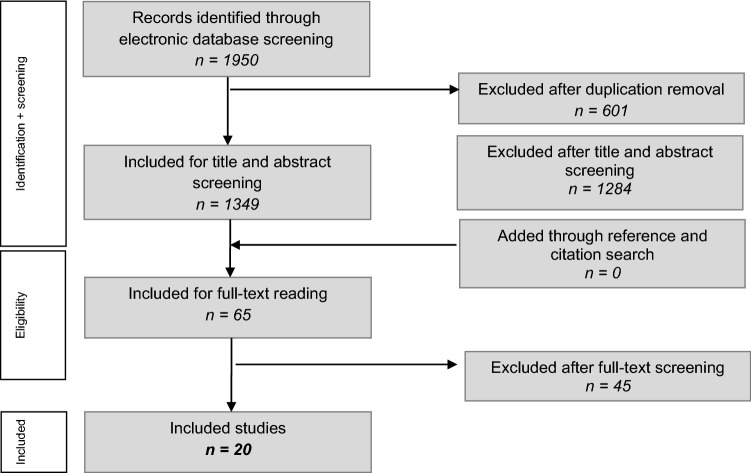
Literature selection algorithms – Preferred Reporting Items for Systematic Reviews and Meta-Analyses (PRISMA)

Clinical studies that investigated the treatment of any type of ankle fracture and also reported findings of OCLs of the ankle were included. Available full-text studies published in English, Dutch and German were eligible for inclusion. No restrictions were set on the publication date nor the age of patients. The exclusion criteria can be found in (Table 1). When necessary, authors were contacted for questions or uncertainties regarding published data. This was also done when additional data was required to be able to execute more detailed data analyses of the included patients. In the case of no response, two reminder e-mails were sent. If there was still no response after three emails, the specific data, and in some cases the whole article, was excluded for (sub)analysis. Independent screening of the title/abstract and full-text of included articles was carried out by two reviewers (H.M. and K.L.). In the case of a conflict, the two reviewers first tried to solve it through a discussion. If this conflict persisted, the judgement of a third investigator (J.D.) was decisive. Studies were not blinded for author, affiliation or source, and no limitation was put on publication status. The literature selection algorithm according to the preferred reporting items for systematic reviews and meta-analyses (PRISMA) is presented in (Fig. 1).

**Table 1:** Study exclusion criteria

Exclusion criteria
Case report studies
< 5 patients included
Data not interpretable
Medical history of ankle surgery
Chronic ligamentous ankle instability
Patient overlap in different studies and no response from corresponding authors after requesting additional information on patient data
Treatment option inappropriately described
Follow-up > 4 years
Level V evidence studies
Animal studies
Cadaveric ankles

### Quality assessment of included studies

To assess the methodological quality of studies the methodological index for non-randomized studies (MINORS) criteria was used [48]. Quality assessment was performed independently by two reviewers (H.M. and K.L.). In the case of a conflict, the judgement of a third, independent investigator (J.D.) was decisive.

### Data extraction

Data were extracted from each included study by one reviewer (H.M.) and cross-checked by one other author (K.L.). Standardised data extraction was performed using a data collection form. Data on study design and study characteristics were extracted and included: year of conduct; number of patients and fractures; patient characteristics (age, sex); type of fracture (according to Danis-Weber classification, Lauge-Hansen classification or other classification/type of fracture); method of OCL diagnosis; OCL incidence; and, lastly, the type of treatment of the OCL [19, 33, 58]. Location and distribution of OCLs were described and classified according to the anatomical osseous location [i.e., talus, tibial plafond, medial malleolus (tibia) or lateral malleolus (fibula)]. If possible and reported, location was specified to an exact location; e.g., anterior, medial, posterior or lateral, and if possible subdivided into anterolateral, anteromedial, posterolateral or posteromedial. If included studies reported on chondral lesions and/or OCL incidence, both incidences were extracted and reported. If included studies reported assessment of OCLs more than 12 months after the initial trauma, we defined the reporting in these studies as ‘late assessment’ and pooled these studies to investigate persistent OCLs after trauma. The before-mentioned studies were excluded from the analysis of the direct assessment of OCL incidence. The intention of this study was to focus mainly on providing a summary of the evidence of incidence rates of OCLs and there was, therefore, less focus on their treatment.

### Terminology

Many derivatives and combinations of chondral, cartilage, defect, lesions and injury were used. Therefore, all reported lesions were considered OCLs and were referred to as OCLs in the results. If studies further classified the lesions as chondral or osteochondral, or if studies used an OCL classification system, stage one of Cheng classification [15, 25], Loomer classification [37], Dipaola classification [23], Outerbridge classification [44] and Berndt and Hardy classification [4] were considered as chondral damage. Subdivision of chondral and osteochondral lesions were reported separately where possible.

### Statistical and data analysis

Reported OCL incidence after ankle fractures was extracted from the original article. If no OCL incidence percentage was reported, we calculated the OCL incidence by dividing the total number of OCLs by the total number of ipsilateral fractures reported in the article. Some studies only reported a specific location (i.e., the talus, tibial plafond, medial malleolus or fibula) but did not specify its sublocation (i.e., anterior, posterior, etc.). If no location of the OCL was reported, only the total number of OCLs was included in the OCL incidence and the OCL incidence was not included in the subanalysis of location. The incidence of OCL per sublocation is based on the number of OCLs per sublocation divided by the total OCL incidence per location. Therefore, it is possible that the number of OCLs in the sublocations will not add up to the total since not all studies reported on the sublocations. The sum of reported OCLs on the sublocations may be greater than the reported OCL incidence after ankle fractures due to the presence of multiple OCLs in a single ankle fracture. If possible and reported, OCL incidence per fracture classification (Danis-Weber and/or Lauge-Hansen classification) was made. Data management and analysis were executed utilizing SPSS. For each variable, frequency distribution, means and standard deviations were calculated. Association between the OCL and fracture type was evaluated by means of an overall *χ*^2^ test. In the case of a statistical significance, post-hoc pairwise comparisons were performed with adjusted significance levels (Bonferroni). Two-sided *p* values of less than 0.05 were considered statistically significant.

## Results

### Search results

The systematic search in PubMed (MEDLINE), EMBASE, CDSR, DARE and CENTRAL yielded 1950 records. After removing the duplicates, 1349 records remained of which title and abstract were screened for relevance. After screening the title and abstract, 1284 records were excluded because the inclusion criteria were not met. The full-text articles of 65 records were screened and assessed for eligibility, resulting in twenty studies eligible for inclusion in the systematic review (Fig. 1). A total of seven authors were contacted to request data according to the inclusion criteria. Additional data from three studies were received, two of which were studies by our co-author. Three [5, 12, 30] of the remaining four studies were excluded for subanalysis of OCL location and subanalysis of association between OCL location and fracture type. After screening and discussion between the first two authors there was overall consensus in all cases of the selection procedure and grading of methodological quality.

### Study and patient characteristics

The study and patient characteristics are summarized in (Table 2). A total of 1707 ankle fractures were included in 1707 patients, and eleven studies (55%) [13, 28, 30, 31, 38, 42, 43, 46, 51, 59, 60] reported on the incidence of the fracture side. Of these studies, 50.5% of the patients had a right ankle fracture and 49.5% a left ankle fracture. No cases of bilateral ankle fractures were reported. Furthermore, the mean MINOR score was 10.5 ± 2.1 (Table 2).

**Table 2:** Study and patient characteristics

Investigator	Year	Study design	Patient (*n*)	Fractures (*n*)	Male (*n*)	Female (*n*)	Mean age (yr)	Age range (± SD) (yr)	Method of diagnosis	Minors
Aktas et al. [1]	2008	Retrospective	86	86	48	38	41.4	14–85 (± 16.7)	Arthroscopical	8
Boraiah et al. [5]	2009	Retrospective	153	153	64	89	51.8	NR	MRI	11
Cha et al. [12]	2015	Retrospective	53	53	33	20	41.0	NR	Arthroscopical	8
Chan et al. [13]	2016	Retrospective	254	254	106	148	45.1	NR	Arthroscopical	12
da Cunha et al. [17]	2017	Retrospective	116	116	66	50	42.7	NR	Arthroscopical	10
Dawe et al. [20]	2014	Retrospective	66	66	41	25	40	NR	Arthroscopical	10
Fuchs et al. [27]	2015	Retrospective	42	42	NR	NR	NR	NR	Arthroscopical	10
Hintermann et al. [28]	2000	Prospective	177	177	86	93	47.2	14–88 (± 18.7)	Arthroscopical	11
Kortekangas et al. [29]	2015	Prospective	48	48	30	18	NR	NR	MRI	11
Kraniotis et al. [30]	2011	Prospective	21	21	13	8	35	NR	CT	13
Lambers et al. [31]	2018	Retrospective	59	59	32	27	37	NR	CT	11
Loren et al. [38]	2002	Prospective	48	48	29	19	35	NR	Arthroscopical	10
Nosewicz et al. [42]	2016	Prospective	100	100	46	54	44	20–77 (± 14)	CT	13
Ono et al. [43]	2004	Prospective	105	105	59	46	45.9	NR	Arthroscopical	10
Regier et al. [46]	2015	Retrospective	99	99	53	46	41.3	NR	MRI	12
Sorrento et al. [49]	2000	Retrospective	50	50	NR	NR	44	NR	Intra-operative	5
Stufkens et al. [51]	2010	Prospective	109	109	61	48	37.4	NR	Arthroscopical	14
Takao et al. [54]	2003	Prospective	92	92	61	31	31.4	18–47 (± 8.7)	MRI + Arthroscopical	11
Ye et al. [59]	2011	Prospective	16	16	13	3	37	NR	Intra-operative	8

*SD* standard deviation; *MINOR* methodological index for nonrandomized studies; *NR* not reported

### Osteochondral lesion incidence directly after trauma

The pooled incidence of OCLs in ankle fractures assessed directly after trauma was 45.1%, as seen by 633 of the 1404 ankle fractures having concomitant OCLs [1, 5, 13, 17, 27, 28, 31, 38, 42, 43, 49, 51, 54, 60]. Thirty nine of these 633 lesions (6.2%) were described as solely chondral lesions according to grade 1 of their corresponding classification [5, 31, 42, 54].

### Location of osteochondral lesions

**Fig. 2:**
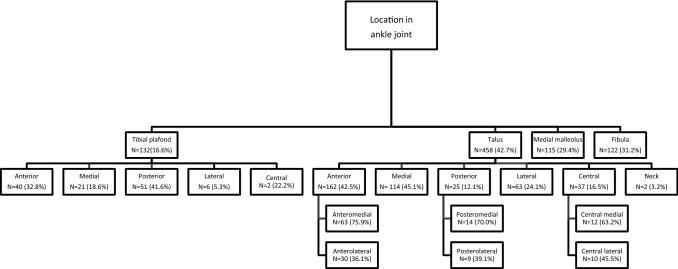
Location of OCL after ankle fractures

Twelve studies reported on the location of the OCLs of which three studies [28, 43, 51] described lesions on the talus, tibial plafond, medial malleolus and fibula. A different four of the twelve studies [13, 27, 31, 38] described lesions on the talus and tibial plafond, and the remaining five studies [5, 17, 42, 49, 60] described lesions solely on the talus. Figure 2 displays the location of OCL per osseous ankle structure. The incidence of OCLs after ankle fractures is 45.1%. Among all of the OCLs, the talus is the location with the highest incidence (42.7%), followed by the fibula (31.2%), medial malleolus (29.4%), and the tibial plafond (16.6%).

### Fracture characteristics

All of the included studies used a type of fracture classification. The Lauge–Hansen was the most utilized fracture classification method and was described in eight of the twenty studies (40%) [5, 12, 17, 29, 43, 49, 54, 60], followed by the Weber classification in three studies (20%) [27, 28, 51]. Three studies used both the Lauge-Hansen and Weber classifications (15%) [18, 38, 42]. Moreover, a combination of the Weber classification with the addition of classification according to isolated medial malleolar fracture, bi- or trimalleolar fracture was used in three studies (10%) [13, 20, 46]. Two studies (10%) [1, 30] classified fractures according to bimalleolar, trimalleolar and distal fibula fracture and one study (5%) [59] described fracture according to the Gustillo open fracture classification.

### Fracture type and OCL incidence and location

**Fig. 3:**
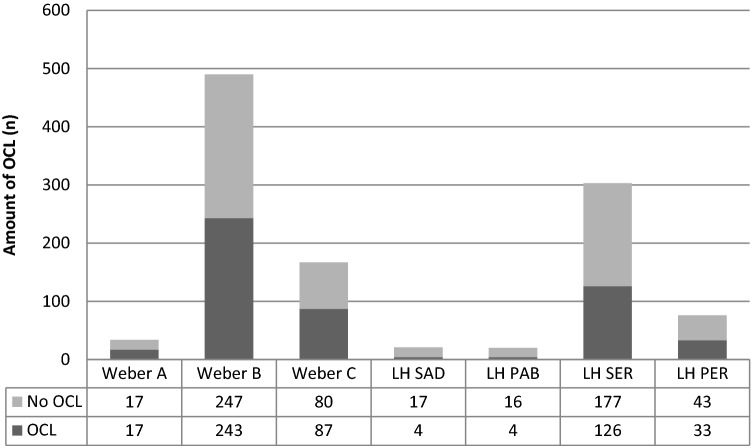
OCL incidence shown per Danis-Weber and Lauge-Hansen classification

Table 3 shows the OCL incidence and location per fracture classification (Danis–Weber [13, 27, 28, 38, 42, 51] and Lauge–Hansen [17, 38, 42, 43, 49, 60]). Furthermore, (Fig. 3) shows the OCL incidence per fracture classification. OCL incidence for Weber classification ankle type fractures was 50.0%, 49.6% and 52.1% for Weber A, B and C, respectively. In Lauge–Hansen ankle type fractures the OCL incidence was 43.4%, 41.6%, 25.0% and 19.0% for Lauge–Hansen supination external rotation (LH-SER), supination adduction (LH-SAD), pronation external rotation (LH-PER), pronation abduction (LH-PAB), respectively. Overall comparison showed no significant difference in OCL prevalence among Weber categories (n.s.), whereas a significant difference was observed among Lauge Hansen categories (*p* = 0.049). Post hoc pairwise comparisons between LH categories (with an adjusted significance level of 0.01) revealed no significant difference (n.s).

**Table 3:** OCL incidence and location per fracture classification

	Total OCL incidence	Talar OCL	Location talar OCL	Tibial plafond OCL	Location distal tibia OCL	Medial malleolus OCL	Distal fibula OCL
Weber A	50.0% (17/34)	48.0% (*N* = 12)	Anterior: 25.0% (*N* = 3) Medial: 33.3% (*N* = 4) Lateral: 41.7% (*N* = 5)	24.0% (*N* = 6)	Anterior: 50.0% (*N* = 3) Medial: 16.7% (*N* = 1) Posterior 16.7% (*N* = 1) Lateral: 33.3% (*N* = 2)	48.0% (*N* = 12)	20.0% (*N* = 5)
Weber B	49.6% (243/490)	40.7% (*N* = 189)	Anterior: 16.4% (*N* = 76) [ant-med 8.1% (*N* = 15), ant-lat 5.4% (*N* = 10)] Medial: 13.8% (*N* = 64) Posterior: 4.1% (*N* = 19) [post-med 6.5% (*N* = 12), post-lat 2.7% (*N* = 5)] Lateral: 6.7% (*N* = 31) Central: 4.5% (*N* = 8) Neck: 1.1% (*N* = 2)	19.8% (*N* = 78)	Anterior: 10.2% (*N* = 22) Medial: 6.0% (*N* = 13) Posterior: 15.3% (*N* = 33) Lateral: 1.4% (*N* = 3)	36.5% (*N* = 70)	47.9% (*N* = 92)
Weber C	52.1% (87/167)	49.0% (*N* = 74)	Anterior: 22.9% (*N* = 33) [ant-med 11.0% (*N* = 8), ant-lat 1.4% (*N* = 1)] Medial: 19.9% (*N* = 30) Posterior: 1.3% (*N* = 2) [post-med 1.4% (*N* = 1), post-lat 1.4% (*N* = 1)] Lateral: 6.6% (*N* = 10) Central: 3.9% (*N* = 2)	43.6% (*N* = 34)	Anterior: 17.9% (*N* = 14) Medial: 6.4% (*N* = 5) Posterior: 20.5% (*N* = 16) Lateral: 1.3% (*N* = 1)	43.3% (*N* = 29)	26.9% (*N* = 18)
LH SER	41.6% (126/303)	28.2% (*N* = 61)	Anterior: 1.3% (*N* = 2) [Ant-med 1.4% (*N* = 1); ant-lat 1.4% (*N* = 1)] Medial: 8.9% (*N* = 14) Posterior: 4.4% (*N* = 7) [post-lat 4.2% (*N* = 7), med-lat 5.6% (*N* = 3)] Lateral: 17.7% (*N* = 28)	5.2% (*N* = 3)	N.A	1.7% (*N* = 1)	10.3% (*N* = 6)
LH PER	43.4% (33/76)	18.4% (*N* = 9)	Medial: 15.6% (*N* = 5) Posterior: 3.1% (*N* = 1) [post-med 100% (*N* = 1)] Lateral: 6.3% (*N* = 2)	5.9% (*N* = 1)	N.A	5.9% (*N* = 1)	0% (*N* = 0)
LH SAD	19.0% (4/21)	5.3% (*N* = 1)	Medial: 100% (*N* = 1)	5.3% (*N* = 1)	N.A	5.3% (*N* = 1)	5.3% (*N* = 1)
LH PAB	25.0% (4/20)	15.8% (*N* = 3)	Lateral: 25.0% (*N* = 1)	0% (*N* = 0)	N.A	0% (*N* = 0)	0% (*N* = 0)

*N.A* Not applicable

### Direct evaluation versus late evaluation of OCLs after ankle fracture

The time-window between trauma and OCL assessment varied between direct evaluation and late evaluation. Whilst fourteen studies [1, 5, 13, 17, 27, 28, 31, 38, 42, 43, 49, 51, 54, 60] evaluated OCL directly after trauma or during primary surgery, six studies [12, 20, 29, 30, 46, 59] assessed OCLs postoperatively, with a mean duration between trauma and assessment varying between 12.3 months and 34.5 months.

In total, 633 OCLs were found in 1404 ankle fractures which were assessed directly after trauma and during primary surgery, thus resulting in an OCL incidence of 45.1%. The OCL incidence of studies which evaluated OCLs more than 12 months after trauma (considered as late evaluation) was 47.5% (144 OCLs in 303 ankle fractures). Direct evaluation and late evaluation in OCL incidence were compared and evaluated by the same assessment mode as shown in (Table 4).

**Table 4:** Mode of assessment of OCL and OCL incidence

Mode of assessment	OCL incidence in direct evaluation	OCL incidence in late evaluation
Ankle arthroscopy	49.5% (406/821) [1, 13, 27, 28, 38, 43, 51]	49.6% (59/119) [12, 20]
MRI	17.0% (26/153) [5]	44.2% (65/147) [29, 46]
CT	12% (19/159) [31, 42]	86% (18/21) [30]
MRI + ankle arthroscopy	70.7% (65/92) [54]	N.A

*N.A* Not applicable

## Discussion

The most important findings of the present review are, firstly, that the OCL incidence directly after ankle fractures is 45.1%. Secondly, it was seen that the most common location of OCLs after an ankle fracture was the talus; it being affected in 42.7% of the reported OCLs.

The OCL incidence ranged from 10 to 88% in the included studies. This broad range could indicate that different definitions, assessment methods and/or staging classifications were used to assess the OCL. Although the term OCL indicates that both the cartilage and the underlying subchondral bone are affected, it is possible that studies included chondral lesions or subchondral cysts under the heading of the definition of an OsteoChondral Lesion. This would give rise to an overestimation of OCL incidence as eight studies did not discriminate between chondral and osteochondral lesions nor did they use a classification system. Staging classification systems are based on the assessment modality; i.e., intra-operative, MRI and CT based assessment. In our study, thirty-nine of the 633 lesions (6.2%) that were assessed directly after trauma were lesions according to grade one of their corresponding classification. On the other hand, an underrepresentation of OCL incidence may also be possible since our search contained ankle fractures and OCL, as well as surgical treatment of all ankle fractures. In addition, no studies were included reporting on the incidence of OCLs after conservative treatment of ankle fractures. It might, however, be important to assess both OCL and solely chondral lesions as a study by Stufkens et al. [51] showed that ankle fractures with OCLs and deep chondral lesions of the talus and distal tibia negatively influence long-term results and is are independent predictors of posttraumatic ankle osteoarthritis.

Methods of diagnosing and assessing OCLs are by radiographs, CT and MRI. Arthroscopic assessment does not discriminate between purely chondral versus osteochondral lesions. This might explain the higher amount OCLs detected through arthroscopy compared to CT and MRI in our study, as both diagnostic methods clearly detect the osseous component [56]. The surrounding soft tissue and cartilage are best visualized by MRI, although that might give an overestimation of osteochondral extent due to bone-marrow oedema [22]. Another possibility of diagnosing an OCL is intra-operatively when utilising an arthroscopically-assisted ankle fracture fixation [14, 34]. Braunstein et al. [7] was, to the best of our knowledge, the first researcher to conduct a RCT in which patients with ankle fractures were randomized into an intervention group (AORIF) or comparison group (ORIF), after which the subjective and functional outcome measurements were evaluated. The 1-year follow-up showed that AORIF lead to good to excellent results in complex ankle fractures [8]. This is in line with previous articles published by Braunstein et al. [6], Liu et al. [36] and Lee et al. [34] However, Fuchs et al. [27] showed no significant functional outcome improvement in patients who underwent AORIF. Another point of discussion is what the surgeon should do when detecting a (osteo)chondral lesion pre- or intra-operatively. Different treatment strategies are possible; ranging from conservative treatment to debridement of the defect, and to bone marrow stimulation and fixation [24]. A recent study by Duramaz et al. [24] found that microfracturing results in significantly more successful clinical results than debridement. Future studies need to focus on identifying the golden surgical treatment option.

### Location

Overall, the most common location of OCLs after ankle fractures was the talus with 42.7% of the OCLs being located here. The medial side was the most common sublocation of the talus, accounting for 45.1% of the talar OCLs, whilst the posterior aspect was the most common sublocation of the tibial plafond OCLs with 41.6% being located here. Only 25 lesions (12.1%) were found on the posterior aspect of the talus. This number could be the result of reporting bias since the OCLs assessed via an arthroscopy were solely performed anteriorly, thus leading to underestimation of posterior-sided talar OCLs. Multiple studies indicate that the occurrence of an OCL on the lateral or medial side of the talus depends on the trauma mechanism, of which medial OCLs usually indicate a mechanism of axial loading and torsional impaction [9, 11, 50]. The systematic review of Verhagen et al. [57] studied the incidence of trauma-associated OCLs and their location, in which they found an incidence of 93% for lateral talar lesions and 61% for medial talar lesions. Comparable findings on the incidence of OCLs in these specific locations were not observed in this review. This could be due to selection bias as not all of the included studies specified the location of talar dome lesions.

### Fracture type and OCL incidence/location

Eight of the included studies used the Lauge–Hansen classification to classify ankle fractures. The rotational impaction factor in ankle fractures is embodied by the Lauge–Hansen classification [33]. Many studies have shown that Lauge–Hansen’s fracture classification has a poor level of agreement among physicians as well as a poor interobserver correlation [2, 10, 55]. This could imply that classifying fractures according to Lauge–Hansen could lead to misinterpretation of the trauma mechanism and its consequences with regards to the analysis of fracture classification and location of the OCL.

Conflicting data have been reported regarding whether there is a significant difference in OCL incidence per fracture type in the Danis-Weber classification. Hintermann et al. [28] reported a significantly higher OCL incidence in patients with a Weber C fracture compared to patients with a Weber B fracture. On the other hand, both Fuchs et al. [27] and Loren et al. [38] found that there was no significant difference in OCL incidence between ankle fractures in the Danis-Weber classification. This is in line with the results of the present systematic review.

A number of previous studies [28, 46] have shown an association between the increase in OCL incidence and the severity of an ankle fracture. Leontaritis et al. [35] found that the number of chondral lesions was significantly associated with more severe ankle fractures, such as Lauge-Hansen PER and SER. In this study, a significant difference in Lauge-Hansen categories (*p* = 0.049) was found. However, post-hoc pairwise comparisons between Lauge-Hansen categories revealed no significant differences. The incidence of OCLs in SER and SAD ankle fractures was 42% and 19%, respectively. This demonstrates that rotational type ankle fractures show a higher incidence of OCLs, and encourages clinicians to be aware of possible OCLs when assessing these types of fractures fractures.

### Direct vs. late assessment

The natural history of OCL and its treatment is described in many studies [3, 11, 26, 38, 39]. However, to the best of our knowledge, no study has been published regarding the healing process of OCLs after ankle fractures. This can be done by assessing the OCL during primary trauma surgery or preoperative radiographs and at the end of follow-up by the same modality. Our study suggests that the natural healing of OCLs is not common as the OCL incidence assessed more than 1 year (range 12.3–34.5 months) after surgery is 47.5% whilst the OCL incidence assessed directly after trauma or during primary surgery is 45.1%. However, it is unclear whether patients had symptomatic OCLs at follow-up and if the assessed OCL was the result of the initial ankle fracture.

Interestingly, when studying the OCL incidence in CT-assessed OCLs, Kraniotis et al. [30] found an incidence of 86% as measured by a CT arthrography scan at late evaluation. This high OCL incidence was most likely due to the mode of assessment, as a CT-arthrography scan evaluates all lesions including cartilage erosions in the form of exposed subchondral bone.

There were a number of limitations in the present review. First, the heterogeneity in the diagnostic assessment methods, the classification systems of OCLs, the terminology, and the locational description of the OCLs. Second, there might have been an underestimation of the OCL incidence because the search excluded studies in which no mentioning was made of OCLs or their incidence after ankle fractures. In addition, there might have been an underestimation of solely chondral lesions since not all assessment methods are capable of detecting these lesions. Another limitation is that the MINORS scores ranged from 5 to 14 out of a total of 16. This was mainly due the retrospective nature of the included studies and the lack of blinding.

The strengths of the present systematic review include the thorough search strategy, the comprehensive quality assessment of the included studies, the data checking by a second reviewer and the extensive contact with authors to retrieve more data as well as asking questions regarding published data and methodology.

The clinical relevance of the present systematic review is that it provides an overview of the incidence of OCLs and their location after ankle fractures. This means that the treating clinical team should pay close attention to the detection of concomitant OCLs in patients with ankle fractures by carrying out adequate pre-operative or intra-operative radiological assessment, or ankle arthroscopy. Hereafter, the team may choose to treat the concomitant intra-articular defect with adequate treatment. The outcomes of the present study will raise awareness to the trauma and orthopedic field of concomitant OCLs in acute ankle fractures and will facilitate the shared-decision making process by enhancing the knowledge on the prognosis and long-term outcomes of acute ankle fractures.

## Conclusion

OCLs in association with acute ankle fractures are frequently seen, as demonstrated by the fact that 45.1% of patients also had an OCL at follow-up until 3 years after the initial trauma. The talus was found to be the specific location with the highest incidence of OCLs (42.7%) and the incidence of OCLs was significantly associated with rotational type ankle fractures.

## Appendix 1

Search terms and results 18-01-2018

**Table:**

Databases		
PubMed, embase (Ovid) cochrane library	Before deduplication	After deduplication
Total	1844	1271

Searches on 18–1-2018:

## PubMed

830 results

(((("Osteochondritis Dissecans[Mesh] OR Osteochondritis dissecans[tiab] OR osteochondrosisdissecans[tiab] OR osteochondrolysis[tiab] OR OCD[tiab] OR OLT[tiab] OR ((osteochondral[tiab] OR chondral[tiab] OR transchondral[tiab] OR cartilage*[tiab]) AND (defect*[tiab] OR lesion*[tiab])))))) AND

((("Ankle Fractures"[Mesh] OR "Ankle Injuries"[Mesh] OR "Ankle Joint"[Mesh] OR "Ankle"[Mesh])) OR (ankle*[tiab] AND (fracture*[tiab] OR injur*[tiab])))

## EMBASE (Ovid)

**Table:**

#	Searches	Results
1	osteochondritis dissecans/ or (osteochondritis dissecans or osteochondrosisdissecans or osteochondrolysis or OCD or OLT).ti,ab,kw. or ((osteochondral or chondral or osteochondral or transchondral or cartilage*) adj3 (defect* or lesion*)).ti,ab,kw	31,561
2	exp ankle fracture/ or exp ankle injury/ or exp ankle/ or (ankle* and (fracture* or injur*)).ti,ab,kw	47,510
3	1 and 2	986

## Cochrane library

CDSR, DARE, CENTRAL: 28 results.

IDSearchHits.

#1MeSH descriptor: (Osteochondritis Dissecans) explode all trees8.

#2osteochondritis dissecans or osteochondrosisdissecans or osteochondrolysis or OCD or OLT:ti,ab,kw (Word variations have been searched)1315.

#3(osteochondral or chondral or transchondral or cartilage*) and (defect* or lesion*):ti,ab,kw (Word variations have been searched)445.

#4#1 or #2 or #3 1738.

#5MeSH descriptor: (Ankle Fractures) explode all trees41.

#6MeSH descriptor: (Ankle Injuries] explode all trees604.

#7MeSH descriptor: [Ankle Joint) explode all trees639.

#8MeSH descriptor: (Ankle) explode all trees465.

#9ankle* and (fracture* or injur*):ti,ab,kw (Word variations have been searched)1576.

#10#5 or #6 or #7 or #8 or #9 2371.

#11#4 and #10 in Cochrane Reviews (Reviews and Protocols), Other Reviews and Trials28.

## Abbreviations

OCL: Osteochondral lesions
Lauge-Hansen SER: Supination external rotation
Lauge-Hansen SAD: Supination adduction
Lauge-Hansen PER: Pronation external rotation
Lauge-Hansen PAB: Pronation abduction
